# Hypogammaglobulinemia in sepsis is not correlated to high circulating angiopoietin-2 levels

**DOI:** 10.1186/cc11957

**Published:** 2013-03-19

**Authors:** U Kovačič, F Starič, M Kmet, M Godnič, R Kapš

**Affiliations:** 1 Faculty of Medicine, University of Ljubljana, Slovenia; 2 General Hospital Novo Mesto, Slovenia

## Introduction

Hypogammaglobulinemia has been frequently found in adult patients with severe sepsis and septic shock. Furthermore, it seems that at least a low serum level of IgM is correlated with higher mortality in sepsis. The mechanisms of hypogammaglobulinemia in septic shock have not yet been explained. It has been hypothesized that outflow of immunoglobulins into the extravascular space due to increased capillary permeability could reduce immunoglobulin serum concentrations. Angiopoietin-2, which directly disrupts the endothelial barrier, is markedly elevated in sepsis and other inflammatory states and its serum level has been correlated with microvascular leakage, end-organ dysfunction and death in sepsis.

## Methods

In the prospective, noninterventional study, we assessed the correlation between the capillary leakage marker angiopoetin-2 and serum levels of IgG and IgM in 41 patients with community-acquired severe sepsis or septic shock on admission. Blood samples were obtained during the first 12 hours after admission to hospital.

## Results

Mean age of patients (17 females) was 70 years. Median APACHE II and SOFA scores at admission were 24 and 11, respectively. The mortality rate was 45%. Thirty-four percent of all patients had level of IgG <6503mg/dl. The median concentration of angiopoietin-2 in the hypo-IgG group was 11,9583pg/ml, which was not statistically different (Mann-Whitney; *P *>0.05) than in the rest of patients with normal levels of IgG (15,688 pg/ml). The concentration of IgM <40 mg/dl was found in only four patients (10%) and all died. Pearson's correlation test showed that the correlation between the concentrations of angiopoietin-2 and IgG (correlation coefficient 0.191) or IgM (correlation coefficient 0.0408), respectively, were not statistically significant (*P <*0.05).

## Conclusion

At present the hypothesis that increased microvascular leakage is responsible for hypogammaglobulinemia in septic patients could not be accepted. Studies on larger number of patients are needed. In addition, it is necessary to further explore other possible mechanisms, such as increased catabolism and consumption of antibodies or inadequate synthesis of immunoglobulins, which could also be responsible for hypogammaglobulinemia in sepsis.
